# Severe Asthma—Perspectives From Adult and Pediatric Pulmonology

**DOI:** 10.3389/fped.2019.00389

**Published:** 2019-10-09

**Authors:** Louise Fleming, Liam Heaney

**Affiliations:** ^1^National Heart and Lung Institute, Imperial College, London and Royal Brompton Hospital, London, United Kingdom; ^2^Centre for Experimental Medicine, School of Medicine, Dentistry and Biomedical Sciences, Institute for Health Sciences, Queens University Belfast, Belfast, United Kingdom

**Keywords:** asthma, children, adults, adherance, type 2 inflammation, biological therapies

## Abstract

Both adults and children with severe asthma represent a small proportion of the asthma population; however, they consume disproportionate resources. For both groups it is important to confirm the diagnosis of severe asthma and ensure that modifiable factors such as adherence have, as far as possible, been addressed. Most children can be controlled on inhaled corticosteroids and long term oral corticosteroid use is rare, in contrast to adults where steroid related morbidity accounts for a large proportion of the costs of severe asthma. Atopic sensitization is very common in children with severe asthma as are other atopic conditions such as allergic rhinitis and hay fever which can impact on asthma control. In adults, the role of allergic driven disease, even in those with co-existent evidence of sensitization, is unclear. There is currently an exciting pipeline of novel biologicals, particularly directed at Type 2 inflammation, which afford the possibility of improved asthma control and reduced treatment side effects for people with asthma. However, not all drugs will work for all patients and accurate phenotyping is essential. In adults the terms T2 high and T2 low asthma have been coined to describe groups of patients based on the presence/absence of eosinophilic inflammation and T-helper 2 (TH_2_) cytokines. Bronchoscopic studies in children with severe asthma have demonstrated that these children are predominantly eosinophilic but the cytokine patterns do not fit the T2 high paradigm suggesting other steroid resistant pathways are driving the eosinophilic inflammation. It remains to be seen whether treatments developed for adult severe asthma will be effective in children and which biomarkers will predict response.

## Introduction

Most children and adults diagnosed with asthma can achieve good symptom control on a low dose of correctly administered inhaled steroids with or without additional controller medication. However, there remains a small but significant proportion with ongoing symptoms and/or frequent attacks despite high intensity treatment. This group consume a high proportion of healthcare resources in terms of treatment costs and hospital admissions, as well as the wider societal costs of time of work and school. Direct healthcare costs are mostly due to prescribed medication for asthma and corticosteroid induced morbidity, particularly in adults (1, 2). In both adults and children, it is essential to firstly confirm the diagnosis of severe asthma in order to implement appropriate management. This article will outline the definition of severe asthma, before exploring the similarities and differences between adults and children in terms of demographics, pathophysiology and management.

## Definition of Severe Asthma

Many terms, including difficult; problematic; therapy resistant and refractory asthma have been used interchangeably with severe asthma. Over the past 20 years national and international organizations have sought to clarify these differences and refine the definitions and nomenclature. The European Respiratory Society (ERS)/American Thoracic Society (ATS) guidelines 2016 (3) set out a framework for the diagnosis of severe asthma in adults and children and more recently the Global Initiative for Asthma (GINA) has produced a useful guide for the diagnosis and management of “Difficult-to-treat” and Severe Asthma in adolescents and adults (4). Only those with a confirmed diagnosis of asthma, prescribed high dose treatment, who have ongoing poor control (or who require a high level of treatment to maintain control) once modifiable factors have been addressed are classified as severe asthma. The diagnosis of asthma is covered elsewhere in this supplement and will not be dealt with further here but identification and addressing poor medication adherence, comorbidities and psychosocial issues is a critical aspect of determining if severe asthma is present and will be discussed below. High intensity standard treatment is generally defined for both adults and children as high dose inhaled corticosteroids (ICS) plus at least one additional controller medication (long acting beta agonist (LABA), theophylline or leukotriene receptor antagonist (LRTA) (5, 6). The definition of high dose ICS is usually stratified by age: children under 5 (or 6) years; between 5 (or 6) to 12 years; and over 12 years. Children and young people (CYP) over 12 years are often included in the adult classification. The rationale for such an approach is likely based on drug licensing studies. Most Phase 3/4 adult studies include adolescent patients but these data are rarely reported separately (or indeed include sufficient adolescents for meaningful subgroup analysis). The definition of “high dose” varies between guidelines. The ERS/ATS and British Thoracic Society (BTS)/Scottish Intercollegiate Guidelines Network (SIGN) guidelines use cut offs of ≥1,000 mcg/day fluticasone propionate (FP) or equivalent for adults and ≥500 mcg/day FP equivalent for children whereas GINA uses lower cut points of >500 mcg FP/day for 12 years and older and >400 mcg for children aged 6–11 years (3, 5, 6). Given the flat dose response curve of ICS, there is little additional benefit from stepping up to very high doses in most patients (7) and therefore the GINA thresholds are probably more appropriate. Furthermore, adolescence is an important time of growth and development and most pediatricians are judicious in their use of very high (i.e., adult) doses of ICS in teenagers, preferring to stick with the pediatric thresholds.

## Prevalence of Severe Asthma

Estimates of the prevalence of severe asthma vary depending on the definition used. It is widely quoted that 5–10% of the asthmatic population have severe asthma, but this will include a significant number of patients with ongoing poor adherence, untreated co-morbidities or mis-diagnosis. In a Dutch study, 17.4% of the adult asthma population had “difficult to control” asthma (poor asthma control despite prescription of high intensity treatment) but after excluding non-adherence and poor inhaler technique, only 3.6% had severe refractory asthma (8). In adults referred with severe asthma, when evaluated systematically with a multi-disciplinary team, high rates of mis-diagnosis and comorbid disease are identified (9, 10). In children the numbers are likely even lower. A study which defined severity on the basis on symptoms, exacerbation frequency and spirometry found that 13% of children with asthma were classified as “severe persistent asthma” (11). However, “difficult to control asthma” would have been a more appropriate label for this group. Of children with difficult to control asthma referred to a specialist severe asthma clinic less than half were assessed as having severe asthma (12). Improvements in assessment of those children, particularly adherence monitoring has seen that figure fall to around 20% (13), suggesting that <2.5% of children with asthma have severe refractory asthma. Severity in pre-school children is even harder to estimate, particularly given the challenges of asthma diagnosis. In the Manchester Asthma and Allergy birth cohort approximately 6% of children with a history of wheeze were classified as severe (14).

## Differentiating Severe Refractory Asthma From Milder Asthma

The umbrella term problematic severe asthma or difficult to treat asthma covers all those with ongoing poor control despite high intensity treatment (5, 15, 16). A significant proportion of this group will have improved control once modifiable factors such as adherence, allergen exposure and psychosocial issues are addressed, and co-morbidities identified and managed appropriately (9, 12, 17). Those with ongoing poor control despite attention to these basics of asthma management are considered to have severe asthma, although it is accepted there is overlap between these groups (18).

## Poor Adherence to Medication

Poor adherence to ICS, for both adults and children is the most common reason for treatment failure and poor asthma control (19, 20). In adults, 65% of patients with “difficult to control” asthma and prone to exacerbation and referred for specialist assessment in the UK, collected <80% of their inhaled preventive medication (21, 22). Medication issues including poor adherence (from prescription records), poor technique and inappropriate device were assessed as contributing to poor asthma control in half of children with seemingly severe asthma referred to a tertiary center (12). Identification of poor adherence to inhaled treatment as the primary clinical problem can be difficult. Physician assessment and patient self-report overestimate adherence when compared with objective or surrogate measures such as prescription records (21, 23). Whilst prescription or dispensing records are helpful, these still overestimate adherence as patients may pick up their inhalers and not use them or use them incorrectly. In children, there is a reliance on parents to request and collect prescriptions; and the child must actually take the medication. A US study which sought to determine if azithromycin or montelukast was an inhaled steroid sparing treatment (MARS trial), was abandoned as most of the children were adequately controlled when adherence was addressed under close medical supervision (24). Objective measures for monitoring inhaled medication are available but have been slow to be implemented in routine clinical care. In both adults and children monitoring adherence to inhaled corticosteroids, aligned with biological response—including suppression of fractional exhaled nitric oxide (FeNO) with monitored treatment—can be used to identify patients with poor prior adherence to inhaled corticosteroids who are likely to achieve substantial benefit from optimized inhaled treatment (13, 23, 25, 26). In addition, this approach can identify those subjects who are unlikely to suppress markers of type-2 biology (FeNO and blood eosinophils) with optimized inhaled treatment alone, and would potentially be candidates for biologic (27, 28). Collectively, these studies have shown that at least 50% of adult and 80% of pediatric patients who would qualify for novel biologic therapies based on their exacerbation history and inflammatory biomarkers are simply not taking regular inhaled anti-inflammatory treatment.

However, having identified that adherence is the primary problem, we still need to define the best intervention to change non-adherent behavior in the medium to long-term and there is unlikely to be a “one size fits all” solution. In adults and children there are a number of factors that can influence adherence, both intentional and unintentional (29): The device must be age appropriate and use demonstrated by an appropriately trained team member (30). Unfortunately, even among healthcare professionals, inhaler technique skills are poor (31). A metered dose inhaler (MDI) should always be used with a spacer in children, and all but the youngest children should be able to use a spacer with mouth piece. In children, an additional complexity is the need to engage parents and caregivers in their child's asthma management. The use of remote directly observed therapy using a Smartphone and associated App has demonstrated that children's inhaler technique is generally inadequate, even when use has recently been demonstrated by a specialist children's asthma nurse (32). Ongoing and continued education is needed, particularly as parents often over estimate their child's technique (33). Furthermore, supervision by parents is often poor; 20% of children aged 7 years are not supervised and by 11 years this figure rises to over 50% (34). Interestingly rates of adherence are equally poor among preschool children; younger school aged children and adolescents (13, 35).

Mobile text messaging in chronic disease management improves adherence rates and simple reminder systems have also been shown to improve inhaled maintenance treatment in asthma, suggesting the gains are potentially large in this population (36–38).

Multiple pharmaceutical companies are now involved with digital technologies and “smart inhalers.” Monitoring of inhaled treatment may potentially become available across a number of inhaler platforms for asthma patients.

## Co-morbidities

In both adults and children, co-morbidities and psychosocial factors, such as atopy, obesity, gastroesophageal reflux (GER), rhinitis, anxiety disorders, depression, and dysfunctional breathing can negatively impact on asthma control, medication adherence, mimic the symptoms of asthma and contribute to the complexity of asthma management in those with both severe and difficult to treat asthma (10, 18, 39). These should be regarded as “treatable traits” which can be identified and targeted as part of a comprehensive disease management approach (40–44). Table 1 [adapted from Porsbjerg and Menzies-Gow (39)] summarizes these extrapulmonary traits and potential management strategies. The prevalence and importance of many of these comorbidities is much better described in adults than children with asthma (39, 53). Identifying and addressing these multiple factors is critically important because factors such as psychological dysfunction can be a major driver of recurrent exacerbation in this population (60).

**Table 1:** Comorbidities in children and adults with severe asthma.

**Clinical Trait**	**Adults**	**Children**	**Management**
Obesity	Patients with severe asthma have a higher BMI than healthy controls and up to 40% are obese (45)	Children with severe asthma have a higher BMI although few children with severe asthma are clinically obese (46)	Weight loss programme; bariatric surgery
Depression and anxiety	Anxiety and depression found up to 27% of patients with difficult asthma (9, 47)	High levels of anxiety in both children with severe asthma and their caregivers	Psychological support
Breathing pattern disorder (BPD); including vocal cord dysfunction (VCD)	BPD common in difficult asthma, 19–52% (39, 48); VCD reported in up to 50% (49, 50)	One study in children found only 6% of children with severe asthma had BPD; this is likely an underestimate (51), prevalence of VCD unknown	Physiotherapy for BPD and EILO; speech and language therapy for VCD; surgery for supraglottic EILO in selected cases
Chronic rhinosinusitis and nasal polyps	Approximately 50% of severe asthma patients have chronic rhinosinusitis; and over 30% nasal polyps (45, 52)	Rare in children; nasal polyps suggest an alternative diagnosis such as cystic fibrosis or primary ciliary dyskinesia	Sinus rinses, nasal steroids, surgery
Allergic rhinitis	Allergic sensitization common in severe asthma (approximately 70–80%; and up to 60% report symptoms of allergic rhinitis (although this is similar in milder asthma) Ref Shaw UBIO	Allergic sensitization and allergic rhinitis common in children (up to 80%) (53)	Anti-histamines, nasal steroids
Obstructive sleep apnoea (OSA)	Common in severe asthma, up to 92% in one cohort (54), 31% in SARP (55)	Reported prevalence of 63% in poorly controlled asthma secondary to adeno-tonsillar hypertrophy (56)	CPAP, weight loss, adenotonsillectomy (children)
Gastro-esophageal reflux	Common in severe asthma, 17–74% (45, 57, 58)	Diagnosed in 20% of children with severe asthma, although unrelated to any measures of asthma severity (46, 59)	Proton pump inhibitors; little evidence to suggest an impact on asthma symptoms

The majority of children (>80%) with asthma are sensitized to at least one aeroallergen, and those with severe asthma are likely to have multiple sensitisations (46, 61). In adults, atopy defined by positivity to skin prick testing or specific IgE measurement in severe asthma cohorts is between 60 and 80% (45, 57, 62), with higher prevalence noted in research cohorts. However, atopy with sensitization to aeroallergens is common in non-asthmatic subjects and in many adults with severe asthma, the role of allergic driven disease even in those with co-existent evidence of sensitization, is unclear. Other atopic conditions such as allergic rhinitis and hay fever are common in children (46) and have been shown to impact on asthma control (63). Chronic rhinosinusitis and nasal polyposis is common in adults, although not children and indeed the presence of nasal polyps in children should be a red flag for an alternative diagnosis such as cystic fibrosis or primary ciliary dyskinesia (9, 45).

Gastro-esophageal reflux (GER) and asthma frequently co-exist in children and adults with asthma (64); however, there is inconsistent evidence of a “cause and effect” relationship between the two GER is associated with asthma symptoms and poor control (65) however, a study of lansoprazole in children with proven GER showed no impact on asthma control (66). A Cochrane review concluded that treatment of GER did not result in an improvement in asthma symptoms in adults or children (67). However, it is possible that non-acid reflux may be important and explain the relationship between symptoms and lack of treatment effect (68).

Obesity is a growing global health problem and can impact on asthma diagnosis, control and exacerbation severity in both adults and children (69, 70). In children the strength of this relationship appears less strong than in adults, although this may in part be because it has been less well**-**studied (71). There are a number of plausible mechanisms for this relationship: deconditioning leading to breathlessness may mimic asthma symptoms leading to an erroneous asthma diagnosis or assessment of control; lung mechanics are altered in obesity with pulmonary restriction and lower functional residual capacity and ventilatory reserve which can have secondary effects on airway smooth muscle shortening during bronchoconstriction; dysynaptic lung growth (increased in lung volume and airway length but not caliber has been shown to be more common on obese children and associated with worse disease severity and poor response to steroids (72–74). Obesity is also associated with systemic inflammation and IL-6 may be a potential mediator of T2 low asthma (and hence steroid resistance) in some obese patients (75). Furthermore, an adult-onset obese female phenotype has been identified with a link to non-eosinophilic airway inflammation (76). Data from small randomized controlled trials show that weight loss improves asthma control, quality of life and lung function but the overall level of evidence of benefit is low (77, 78) and bariatric surgery has been shown to have a positive effect on asthma outcomes (79–81). The relationship between asthma severity and obesity is further confounded by the fact that steroids lead to weight gain. Thus, it can be difficult to untangle whether obesity is driving asthma symptoms (via the mechanisms described above) or whether persistent symptoms and attacks lead to increased steroid use, causing obesity. Furthermore, weight loss is hindered by steroid use.

Anxiety and depression are both associated with increased exacerbation frequency, poorer asthma control and quality of life (60, 82, 83). A negative life event can increase the risk of an asthma attack within a short time of that event and the effect is magnified if there is a background of chronic stress (84, 85). The precise mechanisms underlying the relationship between stress and severity are not known, however an increased T-helper 2 (TH_2_) cytokine response has been associated with higher chronic stress and perceived threat in children (86). It may also be that psychological factors such as anxiety and depression along with other mental health conditions may affect a patient's ability to self-manage their asthma and impact on other behaviors such as smoking and poor adherence.

Dysfunctional breathing (DB) is common in adult patients with severe asthma with a reported prevalence of 19–52% (39, 48). There is good evidence that breathing retraining can improve asthma control and quality of life (87, 88). Much less is known about DB in children. A single study assessing dysfunctional breathing in a pediatric severe asthma clinic found that only 5.4% were found to have a breathing pattern disorder (51). It is likely that DB in under-recognized in children and in the broader population of children with difficult to treat to asthma the prevalence is likely much higher. A more comprehensive review of this topic can be found elsewhere in this supplement.

## Smoking

Although active cigarette smoking does not appear to be related to the development of adult onset asthma, it is associated with asthma severity, frequent exacerbations and lung function decline (89, 90). Active smoking has been shown to reduce expression of histone deacetylase (HDAC2) via phosoinositide-3-kinase activation, leading to reduced glucocorticoid sensitivity (91). The same has also been found in children with severe asthma exposed to second hand smoke (92).

## The Pathobiology of Severe Asthma and Asthma Phenotypes

Both pediatric and adult severe asthma are characterized by heterogeneity in clinical expression and underlying pathobiological features (Table 2).

**Table 2:** Comparison of phenotypes in adults and children with severe asthma.

**Phenotype**	**Description**	**Adults**	**Pediatrics**	**Therapy**
T2 high	High levels TH_2_ cytokines (IL4, IL5, and IL13); biomarkers include airway and blood eosinophils, FENO, and periostin	More severe disease with higher risk of asthma attacks	Children with severe asthma are predominantly eosinophilic, although TH_2_ cytokines may not be elevated	Steroid sensitive, anti IgE or anti IL4, IL5, or IL13
T2 low	Low levels of eosinophils, often associated with airway neutrophilia	Patients tend to be older; associated with low FEV_1_ and air trapping, poor steroid response	Little evidence for T_2_ low asthma in children; airway neutrophils may be protective; airway neutrophilia associated with infection	Macrolide antibiotics may have some benefit
Persistent airflow limitation	Low FEV_1_ or FEV_1_/FVC post bronchodilator, post steroid trial	40–60% with severe asthma, older age, longer asthma duration, smoking (93)	25% of children with severe asthma have PAL; associated with increase in airway smooth muscle (94–96)	Optimize long acting bronchodilators, down titrate steroids to lowest dose to control symptoms
Atopic	Sensitisation to aeroallergens and elevated IgE	Associated with early onset asthma persisting into adulthood	Most children are atopic and have co-morbid atopic diseases	Steroid responsive; T_2_ targeted therapies as above

In contrast to adults, where a female preponderance is very consistently seen, there is a male preponderance in childhood (47, 97). The increasing proportion of women with severe asthma through the life course is particularly seen in adolescence and around the time of the menopause suggesting a link with female sex hormones (98).

## T2 High and T2 Low Asthma

In 1958, Dr. Harry Morrow Brown published a seminal paper in the Lancet, which demonstrated that the clinical response to corticosteroids in adults with chronic persistent asthma was associated with the presence of eosinophils in sputum (99). This study was performed after the United Kingdom Medical Research Council subcommittee on clinical trials in asthma reported no advantage of cortisone acetate in the treatment of chronic asthma, which was a result which many, including Dr. Morrow Brown, found surprising (99). This outcome may have been due in part to the study design and reported outcomes but also in part to the inclusion of a heterogeneous patient population with a clinical diagnosis of asthma. Many years later, a study in adults using sputum samples induced with hypertonic saline also demonstrated that patients with asthma and a sputum eosinophil count ≥3% had a greater response to inhaled corticosteroids (ICS) compared to those with an eosinophil count <3% (100). Woodruff et al. (101) subsequently demonstrated that an IL-13 derived epithelial gene signature (periostin, serpin B2, and CCLA1) was associated with airway eosinophilia and upregulation of T-helper 2 (TH_2_) cytokines IL-5 and IL-13 but importantly this was only seen in 50% of asthmatics who had withdrawn ICS. Further, reintroduction of ICS was again associated with an improvement in lung function in subjects with evidence of eosinophilia and upregulation of type-2 cytokines, but which importantly was not seen in those subjects without evidence of airway type-2 inflammation/eosinophilia. The authors coined the terms TH_2_-high and TH_2−_low asthma and reinvigorated the debate about non-eosinophilic asthma and importantly asthma, which is not responsive to ICS. The classical paradigm was that this process was predominantly driven by TH_2_ cells however it is now recognized that there are other cellular sources of these cytokines and innate lymphoid cells and mast cells are potential sources of these pro-inflammatory cytokines (102, 103).

Asthma in children is predominantly an atopic disorder. Children with severe asthma are characterized by multiple aeroallergen sensitization, food allergy, high exhaled nitric oxide and progressive loss of lung function throughout childhood (47, 97, 104). Childhood severe asthma is predominantly eosinophilic with increased luminal (bronchoalveolar lavage) and tissue eosinophils (105). However, inflammatory phenotypes based on sputum are not stable in children (106). Furthermore, bronchoscopic studies have demonstrated an absence of TH_2_ cytokines (105, 107) suggesting that airway eosinoplhia in children is mediated by relatively steroid resistance pathways. IL-33 remains elevated despite maximal steroid therapy (108, 109) and increased levels of innate lymphoid cells (ILCs) in children with severe asthma suggests a possible therapeutic target (110). Few studies in children have targeted eosinophils. The only study in children that based management on sputum eosinophils did not show a significant reduction in exacerbations (111), in contrast to benefit demonstrated in adult studies as described above (112).

Bronchoscopic studies in adults have provided rich evidence for underlying pathobiology of adult severe asthma and potential endotypes. Mechanistic studies in children are hampered by ethical constraints: samples from healthy controls have to be opportunistically collected from those having bronchoscopy for upper airway problems or a general anesthetic for an unrelated procedure and it is not possible to repeat bronchoscopies to assess the airway response to an intervention or challenge.

Recent data suggests that steroid resistant type-2 biology in adults is associated with older patients. There is likely to be a degree of overlap but our understanding of inhaled steroid resistant disease has been hampered by our prior failure to measure effective drug delivery in mechanistic studies (25, 113).

## Non-T2 Mechanisms in Severe Asthma

A study using data form the National Asthma Research Programme in the United States demonstrated that subjects with mild to moderate asthma and no evidence of sputum eosinophilia (defined as sputum eosinophil count <2%) had no improvement in lung function with ICS but showed evidence of a bronchodilator response consistent with asthma (114). This study also identified that in ICS naive subjects 47% have no evidence of sputum eosinophilia in serial samples taken at various points over a 1-year period but this proportion increased to 72% in subjects currently taking ICS treatment. Taken together, these data suggests that substantial groups of patients with mild and moderate asthma have little evidence of type-2 cytokine driven airway eosinophilia (T2-low asthma) and by extension are likely to have little response to either initiation or escalation ICS treatment. Unfortunately, many patients who are unresponsive to inhaled steroids have their doses relentlessly increased, with no clinical benefit but substantial risk of harm.

McGrath et al. (114) also reported that the patients with non-eosinophilic mild asthma had less bronchial hyperresponsiveness than those with airways eosinophilia and Arron et al. (115) also demonstrated an inverse relationship between an IL13-high gene signature (T2-high asthma) and bronchial hyperresponsiveness suggesting that T2-low/non-eosinophilic disease is associated with less physiological abnormalities. This is supported by data which suggest that non-eosinophilic asthma is associated with lower risk of asthma exacerbation and there is strong evidence that surrogate biomarkers of type-2 airways inflammation (blood eosinophil count, FeNO, serum ECP, and serum periostin) are all prognostic biomarkers for exacerbation (116–118). In addition, elevated blood eosinophil count and FeNO have consistently been associated with a higher exacerbation rate in the placebo arms of clinical trials in patients with severe asthma unselected on the basis of a pre-stipulated biomarker threshold (119–121) This would suggest that within severe asthma populations, defined using usual diagnostic and treatment criteria, there are subjects with little evidence of active T2-driven disease (type-2 biomarker low), who are persistently symptomatic and defined as uncontrolled asthma, but who have a comparatively low exacerbation risk. However, it is important to comment that severe asthma exacerbations still occur in these symptom high type-2 biomarker low patients albeit at a much lower rate.

It is recognized that ICS will down-regulate type-2 inflammation and related biomarkers (122, 123) and is thus an important confounder in subjects with more severe asthma on ICS treatment raising two important issues—firstly, whether true T2-low asthma exists in more severe asthma, when corticosteroids are down titrated and secondly, what is the mechanism of persistent symptoms in subjects with asthma when type-2 corticosteroid responsive asthma is not present. As discussed above, “true” T2-low asthma appears to be relatively common in mild asthmatic subjects but is generally associated with mild symptoms, less bronchial hyperresponsiveness and a lower risk of asthma exacerbation compared to subjects with T2-high disease. Intuitively, it seems improbable that this group of patients would be progressed to high dose ICS treatment, however if symptoms are persistent and not ICS responsive as appears to be the case, it is possible that some of these patients may be escalated to high intensity treatment to try and improve their symptoms. The issue of whether true T2-low asthma exists in severe asthma is currently being explored in the RASP-UK programme, where patients are having corticosteroids “down titrated” if their biomarkers of T2-driven disease are low (124).

The neutrophilic phenotype is not seen consistently in children (106) and there is evidence to suggest that airway neutrophils may be protective (125) and very little is known about T2 low asthma in children (if it exists at all).

Non-atopic clinical phenotypes described in adults including those associated with sensitivity to non-steroidal anti-inflammatories and nasal polyposis are rare in childhood.

## Airway Remodeling

Airway remodeling starts early in childhood asthma. Although no differences in reticular basement membrane (RBM) thickness were seen in infants with respiratory symptoms with or without evidence of reversible airflow obstruction at a median 1 year of age (126) by a median of 3 years there were significant differences between those with troublesome wheeze and healthy controls (127) and by school age the changes were consistent with those seen in adult severe asthma (128). Interestingly, only increased airway smooth muscle in pre-schoolers predicted the persistence of asthma at school age (129). Almost a quarter of children with severe asthma have persistent airflow limitation (PAL), defined as post systemic post-bronchodilator z score ≤ −1.96 for FEV_1_ (94, 97). In adults this rises to almost 50% and is associated with older age, longer duration of asthma symptoms and airway eosinophilia (93). In children there appears to be no association between PAL and eosinophilic inflammation although there is evidence of an increase in airway smooth muscle surface area and mass in those with PAL (95, 96).

## Management

As previously discussed, the first stage of management is the confirmation of the diagnosis of severe asthma and the comprehensive assessment and modification of co-morbidities and factors driving persistent symptoms and asthma attacks. For who remain poorly controlled following this assessment and remain on high intensity conventional treatment (high dose ICS as defined above, plus additional add on treatments including LABA, LRTA or theophylline or maintenance OCS) there is an exciting pipeline of targeted treatments which predominantly target the type-2 cytokine axis including IL-4, IL-5, and IL-13 causing IgE production and eosinophilic inflammation (130, 131).

Omalizumab, the monoclonal antibody to IgE, was the first biologic licensed in the United Sates (US) in 2003 in adults and children (>12 years) and by 2009 it was approved in the US and Europe for all patients with asthma from the age of 6 years. The last 3 years have seen an acceleration in the number of Phase 2 and 3 studies and the licensing of new drugs. The choice of biologic depends on the patient's age, weight, IgE evaluation (both total and specific), degree of eosinophilia and route of administration. The Global initiative for Asthma (GINA) has recently produced a pocket guide to aid the physician in determining the most appropriate treatment for the individual (4).

## Omalizumab

Omalizumab is a recombinant antibody to IgE that diminishes free IgE levels and prevents IgE binding to mast cells and basophils, thus inhibiting degranulation and release of proinflammatory mediators. Studies in adults and children have demonstrated benefit, particularly in terms of reduction in exacerbations (132). The dose is based on IgE level and weight. Approximately 25% of children have an IgE level outside the licensed range and for those at the upper end of the range it may entail 2 weekly injections (97) and additionally adults may not qualify without sensitization to an aeroallergen. The frequency of these scheduled visits must be balanced against a reduction in attacks and hence unscheduled time off school and work. In children omalizumab has also been used seasonally to attenuate the September peak in asthma attacks (133) and this approach showed greatest utility in children on the higher treatment steps. In a randomized withdrawal study, after long-term treatment with omalizumab, patients on omalizumab did better than those randomized to placebo suggesting continuation of omalizumab results in continued benefit and does not support a disease modifying effect of this treatment (134).

## Drugs Targeting IL-5—mepolizumab, Reslizumab, and Benralizumab

Mepolizumab is a humanized monoclonal antibody to IL-5 that inhibits eosinophilic maturation and activation. The DREAM and MENSA studies recruited patients with recurrent asthma exacerbations and evidence of eosinophilic inflammation despite high doses of inhaled glucocorticoids and demonstrated a reduction in asthma attacks in those randomized to mepolizumab (135, 136). In subjects with increased blood eosinophil levels, greater efficacy was seen with additional improvements in lung function and symptoms. Although adolescents were recruited for the licensing studies the data were not reported separately and there are no specific pediatric randomized controlled trials. The results of an open label pharmacokinetics and pharmacodynamics study in children aged 6–12 years are awaited (137). Despite this, mepolizumab has recently been licensed by the European Medicines Agency (EMA) for use in patients with severe asthma from 6 years of age. Two further drugs targeting the IL-5 axis have recently been licensed by the EMA for adults: reslizumab and benralizumab. Reslizumab has a similar mode of action to mepolizumab. It binds to IL-5 thus reducing IL-5 mediated maturation and trafficking of eosinophils from the bone marrow as well as inhibiting mediator release and eosinophil apoptosis. It is given by 4 weekly intravenous infusion and demonstrated a significant reduction in exacerbations in the reslizumab arm compared to placebo (138). It is licensed by the Food and Drug Administration (FDA) and EMA for use in adults only and notably studies using a fixed sub-cutaneous dose of 110 mg were negative (139, 140). Benralizumab binds to the IL-5 receptor and in addition to inhibiting growth and activation of eosinophils benralizumab also binds to the Fc receptor on natural killer cells and induces antibody dependent cell mediated cytotoxicity leading to apoptosis of eosinophils. In clinical trials benralizumab led to decreased exacerbations, improved lung function and enhanced quality of life (141, 142). It is theoretically more effective than other IL-5 blockers which allow IL-5 independent migration of eosinophils into the tissue, although superior efficacy was not evident in clinical trials to date and the results of targeting the IL-5 axis seem broadly consistent with reduced exacerbation rates (rescue OCS ≥ 3 days) by approximately 50% and more modest improvements in symptoms and asthma-related quality of life measured and lung function (143). Benralizumab and mepolizumab have also been shown to reduce oral corticosteroid dose compared to placebo with a parallel reduction in exacerbation rate in adult subjects with severe asthma on maintenance oral corticosteroids (144, 145). It also has the advantage of being given every 8 weeks after the initial three, four weekly injections. The FDA have licensed benralizumab for use as an add on treatment for patients with eosinophilic asthma aged 12 years and above. In Europe it has been licensed by the EMA for use in adults only.

## Dupilumab

Dupilumab binds to the IL-4 receptor-α blocking signaling of IL-4 and IL-13, thus reducing IgE production and eosinophil recruitment. Phase 3 studies have demonstrated a significant reduction in severe exacerbations (47.7% reduction for 200 mg dupilumab compared to placebo, *p* < 0.001) and additionally demonstrated lung function improvement which was evident early after treatment initiation (at week 12, FEV_1_ increased 0.14 liters compared to placebo, *p* < 0.001) (120). Dupilumab treatment also resulted in a significant reduction in OCS in adult subjects with severe asthma on maintenance OCS treatment (70.1% with dupilumab vs. 41.9% in the placebo group (*p* < 0.001) with a 59% lower exacerbation rate than placebo and mean increase in FEV1 of mean 0.22 liters more than placebo (146). It was licensed in October 2018 by the FDA and in May 2019 by the EMA for use in patients with severe eosinophilic asthma from 12 years of age. It also has a license for treatment of severe atopic eczema and will clearly be a useful treatment in patients with both conditions.

It is disappointing that no data from pediatric specific randomized controlled trials have been presented for mepolizumab, reslizumab, benralizumab or dupilumab and there has been no separate analysis of the adolescents recruited for these studies (likely because of insufficient numbers for meaningful subgroup analysis). Given the challenges recruiting to the MARS trial as discussed above (24), and the inference that there are relatively small number of children who have uncontrolled asthma when adherent with standard of care inhaled treatment, it is acknowledged that the feasibility of any such study is challenging and the need for international collaboration essential (147, 148). None-the-less the majority of these drugs have been licensed by the FDA and EMA for 12 years and older and in the case of mepolizumab by the EMA for use from the age of 6 years. In view of the limited add on choices for children with severe asthma, the addition of an increasing number of biologics is to be welcomed. It will be important to use “real-world” outcome data to identify if similar efficacy is seen in pediatric cohorts and to identify markers of response (149). In addition, it has become clear that there is substantial overlap in the populations for these new biologic therapies and randomized pragmatic head to head comparisons between the various biologics will potentially identify which patients should be treated with each drug (149, 150).

## Summary

There is heterogeneity both within and between pediatric and adult severe asthma. Although there is overlap between the two there are a number of differences in clinical expression and disease mechanisms in children and adults. The impact of the growing lung and developing immune system is likely to account for some of the observable differences however, much remains poorly understood, including the natural history of asthma; adult onset vs. childhood onset disease; and the impact of early life influences (including in utero). Studies such as the Unbiased Biomarkers for the PREDiction of respiratory outcomes (U-BIOPRED) marks a step change in asthma research. Cohorts of preschool, school aged and adults with severe asthma (45, 46) were recruited to this multi-omics study which aims to define phenotypes and endotypes of severe adult and pediatric asthma and preschool wheeze and compare these groups across the life course. In the meantime, there remain a number of unmet needs in the management of severe asthma. Expensive biologics are available to only the minority of patients with severe asthma globally; there is an ongoing disease burden both in terms of asthma related morbidity and mortality and corticosteroids, the mainstay of treatment for many, have an unacceptable side effect profile. Finally, there is the absence to date of any disease modifying treatments. The window of opportunity for such an intervention is likely to lie in very early childhood and therefore to improve the care of all patients with severe disease, there needs to be investment and a focus on research in young children.

## Author Contributions

All authors listed have made a substantial, direct and intellectual contribution to the work, and approved it for publication.

### Conflict of Interest

The authors declare that the research was conducted in the absence of any commercial or financial relationships that could be construed as a potential conflict of interest.
